# Glioma Mimics: Magnetic Resonance Imaging Characteristics of Granulomas in Dogs

**DOI:** 10.3389/fvets.2019.00286

**Published:** 2019-08-28

**Authors:** Lauren Diangelo, Aaron Cohen-Gadol, Hock Gan Heng, Margaret A. Miller, Devon W. Hague, John H. Rossmeisl, R. Timothy Bentley

**Affiliations:** ^1^Department of Veterinary Clinical Sciences, College of Veterinary Medicine, Purdue University, West Lafayette, IN, United States; ^2^Department of Neurological Surgery, Indiana University School of Medicine, Indianapolis, IN, United States; ^3^Department of Comparative Pathobiology, Purdue University, West Lafayette, IN, United States; ^4^Department of Veterinary Clinical Medicine, College of Veterinary Medicine, University of Illinois, Urbana, IL, United States; ^5^Department of Small Animal Clinical Sciences, Virginia-Maryland College of Veterinary Medicine, Blacksburg, VA, United States

**Keywords:** brain, canine, fungal, granulomatous meningoencephalomyelitis, pseudotumor

## Abstract

Granulomas can “mimic” gliomas on magnetic resonance imaging (MRI) in human patients. The goal of this retrospective study was to report canine brain granulomas that were consistent with glioma based upon MRI, report their histologic diagnosis, and identify MRI criteria that might be useful to distinguish granuloma from glioma. Ten granulomas, initially suspected to be glioma based on MRI, were ultimately diagnosed as granulomatous meningoencephalomyelitis (*n* = 5), infectious granulomas (*n* = 3) or other meningoencephalitis (*n* = 2). Age was 1.6–15.0 years and two dogs were brachycephalic breeds. MRI characteristics overlapping with glioma included intra-axial, heterogeneous, T2-weighted hyperintense, T1-weighted hypointense to isointense mass lesions with contrast-enhancement. Signals on fluid attenuation inversion recovery, gradient echo and diffusion weighted imaging also matched glioma. Peri-lesional edema and mass effect were toward the high end of findings reported for glioma. MRI characteristics that would be considered unusual for glioma included dural contact (*n* = 4), T2-hypointensity (*n* = 2), concomitant meningeal-enhancement (*n* = 9), and minor changes in the contralateral brain (*n* = 2). Cerebrospinal fluid analysis revealed albuminocytological dissociation or mild pleocytosis. These cases show that granulomas can “mimic” glioma on canine brain MRI. In individual cases, certain MRI findings may help increase the index of suspicion for granuloma. Lack of pronounced cerebrospinal fluid pleocytosis does not exclude granuloma. Signalment is very useful in the suspicion of glioma, and many of these dogs with granuloma were of ages and breeds in which glioma is less commonly seen.

## Introduction

In human medicine, many intracranial inflammatory processes may have magnetic resonance imaging (MRI) characteristics that suggest glioma. A common example is sarcoid granuloma due to sarcoidosis (1–4). Sarcoid granulomas are often initially misdiagnosed as glioma (1–4), or in other locations as meningioma (5, 6) or trigeminal schwannoma (7). Other inflammatory syndromes with MRI characteristics suggestive of glioma include fungal granuloma (8), eosinophilic granuloma (9), foreign body reactions to surgical (10), or embolization material (11), or inflammatory pseudotumor (12). These reports all state that the granuloma “mimicked” or “masqueraded” as a glioma on MRI (1–4, 8–11).

Similarly, in dogs fungal granuloma can be mistaken for glioma on MRI (13, 14) and both should be included in the differential diagnosis for intra-axial masses with peripheral contrast-enhancement (15, 16). Other granulomas with a neoplasm-like MRI appearance include cholesterol granuloma (17), granulomatous *Acanthamoeba* encephalitis (18), inflammatory pseudotumor (19), and the focal form of granulomatous meningoencephalomyelitis (GME) (20). In GME, meningeal changes are expected histologically but are rarely apparent on MRI (21). While GME is often associated with multifocal lesions at the histological level, on MRI a singular lesion is observed in over a third of cases (21). The cerebral white matter is a prime location for multiple perivascular cuffs to coalesce into a singular granuloma (22), adding to the MRI difficulty in distinguishing between GME and glioma.

Accordingly, human granulomas are known to mimic glioma on MRI. In dogs, more information is required regarding which diseases might cause glioma-like MRI lesions, and how these might be distinguished from glioma (23). The goal of this study was therefore to report canine brain granuloma “glioma-mimics” on MRI and their histologic diagnosis, and to identify any MRI features that might allow accurate differentiation of granuloma from glioma. We hypothesized that granulomas would share MRI characteristics with glioma such as a heterogeneous T2 signal and mass effect, but that some cases of granuloma would have characteristics such as concomitant meningeal contrast-enhancement that would be considered atypical for glioma.

## Materials and Methods

### Inclusion Criteria

A retrospective study was performed. Medical records at the veterinary teaching hospitals of the Purdue University and Virginia-Maryland Colleges of Veterinary Medicine were searched for dogs that had brain MRI (September 2008–September 2018). A histological diagnosis of a granuloma (surgical biopsy or necropsy) at the Purdue University or Virginia-Maryland College of Veterinary Medicine was required for inclusion. It was also required that the radiology report included glioma (or a comparable reference to intra-axial neoplasia) as a principal differential diagnosis. All infectious and non-infectious etiologies of granuloma were included.

### MRIs

The MRIs were jointly reviewed by two investigators (LD, RTB) using standardized criteria developed from similar previous studies (24, 25). These included location, origin, dural contact, lesion number, mass effect, intensity, and homogeneity of signals [T1-weighted (T1W), T2-weighted (T2W), fluid attenuation inversion recovery (FLAIR), gradient echo (GRE), diffusion weighted imaging (DWI)], peri-lesional edema, lesion margins, the strength and pattern of contrast-enhancement, and meningeal contrast-enhancement.

### Medical Records Review

Information collected from the medical record included signalment, neurological deficits, and results of diagnostic tests including cerebrospinal fluid (CSF) analysis, serology and histology. The radiology report from the time of the MRI was reviewed for additional diseases included in the principal differential diagnosis, and any diseases mentioned as secondary possibilities.

## Results

### Cases, Histology, and Ancillary Diagnostics

Ten dogs met the inclusion criteria. Ages were 1.6–15.0 years of age (median, 6.3 years). Sexes were female spayed (*n* = 6), male neutered (*n* = 3), or male intact (*n* = 1). Breeds represented were Labrador retriever (2), mixed (2) and one each of Airedale, Border collie, Doberman pinscher, Golden retriever, Pekingese, and Pug (Table 1).

**Table 1:** Signalment, MRI and CSF findings, and final diagnosis for 10 intracranial granulomas.

**Case**	**Age (years)**	**Breed**	**Main MRI lesion**	**Meningeal enhancement on MRI**	**Contralateral MRI lesions**	**CSF analysis**	**Diagnosis**
1	4.9	Pug	Heterogeneous intra-axial temporal mass	None	None	AC dissociation	GME
2	3.7	Airedale terrier	Heterogeneous intra-axial parieto-occipital mass	Adjacent meninges; single leptomeningeal line	None	NP	GME
3	4.3	Labrador retriever	Heterogeneous intra-axial occipital mass	Mass contacts enhancing tentorium cerebelli	None	NP	Fibrosing neutrophilic meningitis
4	9.0	Labrador retriever	Heterogeneous mixed (intra-axial and extra-axial) frontal mass	Mass contacts adjacent enhancing dura mater	None	AC dissociation	Lymphoplasmacytic meningoencephalitis
5	4.5	Mix	T2-isointense intra-axial parietal mass with a T2-hypointense focus	Adjacent meninges (cerebral and falx cerebri)	None	NP	Fungal granuloma, GMS-positive hyphae
6	1.6	Doberman pinscher	T2-hypointense cerebellar intra-axial mass	Mass contacts enhancing tentorium cerebelli	None	Mononuclear pleocytosis (TNCC 31)	*Blastomyces* granuloma
7	7.6	Mix	Heterogeneous intra-axial temporal mass with pinpoint GRE signal voids	Mass contacts adjacent dura mater; single leptomeningeal line	None	Mixed pleocytosis (TNCC 16)	GME
8	9.0	Golden retriever	Heterogeneous intra-axial temporal mass	Ipsilateral cerebral hemisphere (mild)	None	AC dissociation	Infectious encephalitis
9	15.0	Border Collie	Heterogeneous intra-axial temporo-parieto-occipital mass	Bilateral telencephalon, midbrain and ventral brainstem (patchy)	Focal parenchymal enhancement; patchy bilateral meningeal enhancement	Mononuclear pleocytosis (TNCC 34)	GME
10	11.0	Pekingese	Heterogeneous intra-axial temporo-thalamic mass with a T2-hypointense focus and multifocal GRE signal voids	Ipsilateral cerebral hemisphere (patchy)	Focal parenchymal T2-hyperintensity; focal contralateral meningeal enhancement	NP	GME

AC, albuminocytological; CSF, cerebrospinal fluid; GME, granulomatous meningoencephalomyelitis; GMS, Grocott's methenamine silver; GRE, gradient echo (T2^*^-weighted imaging); MRI, magnetic resonance imaging; NP, not performed due to concern for raised intracranial pressure; TNCC, total nucleated cell count (cells per μL).

There was a 4 day to 8 week (median, 4 weeks) history of neurological clinical signs. One case had recently had splenectomy for splenic hematoma. In all other cases, there was no historical evidence of extracranial disease. Six cases had MRI elsewhere and were specifically referred by a veterinary neurologist for surgery of a suspected intracranial neoplasm. Incidental physical examination findings included stridor (Pug), two small subcutaneous masses (suspect lipomas; 9 year old Labrador retriever), or pigmentary keratitis (Pekingese). Physical examination was otherwise unremarkable. Complete blood counts and serum chemistries were within normal limits except lymphopenia or stress leukogram (*n* = 3), mild anemia (*n* = 2), very mild elevations in amylase and lipase (*n* = 1), and increased liver enzyme activity consistent with phenobarbital therapy (*n* = 1). Chest radiographs were normal (*n* = 5), as were the results of various other investigations [e.g., abdominal radiographs/ultrasound (*n* = 3), coagulation times (n = 2)]. Following MRI, eight cases improved or normalized in response to anti-inflammatory steroids.

Neurological deficits referable to the forebrain predominated, including thalamocortical visual deficits (*n* = 6), seizures (*n* = 5), mentation or behavioral change (*n* = 5), proprioceptive deficits (*n* = 3), circling (*n* = 2), and nasal hypalgesia (*n* = 1). Signs in forebrain lesions with secondary brain herniations or in cerebellar lesions included head tilt (*n* = 2) and proprioceptive (*n* = 1) or vestibulocerebellar (*n* = 1) ataxia.

Histological diagnoses were GME (*n* = 5), infectious granuloma (*n* = 3), lymphoplasmacytic meningoencephalitis (*n* = 1), or focal fibrosing and neutrophilic meningitis (*n* = 1). The diagnosis was acquired from surgical biopsy (*n* = 7) or necropsy (*n* = 3).

The three infectious cases included one *Blastomyces* granuloma and one granuloma with Grocott's methenamine silver-positive hyphae of an unidentified fungal species. In the third infectious case, stereotactic biopsy revealed marked chronic suppurative encephalitis and moderate chronic suppurative meningitis suggestive of infection. Micro-organisms were not identified, by histology or by additional testing (below). This dog was presented for seizures. No additional neurological deficits developed during therapy with doxycycline, clindamycin, enrofloxacin, phenobarbital, and tapering prednisone, and the inter-ictal interval improved (9 month follow-up).

The surgical findings in the lymphoplasmacytic meningoencephalitis case were a mass lesion extending from the underside of the dura mater to the depth of peri-ventricular cerebral white matter. Histologically, inflammation extended from meninges to cerebral tissues. There were areas of fibroplasia reminiscent of an inflammatory pseudotumor (12, 19), and Mason's trichrome staining confirmed some fibrous collagen. The dog with a histological diagnosis of focal fibrosing and neutrophilic meningitis had distinctly improved while receiving anti-inflammatory corticosteroids for 2 weeks pre-operatively. At surgery, discolored cerebral parenchyma and grossly normal dura mater were submitted for histology.

Negative infectious disease testing of the 10 cases included: various mycosis testing in eight cases (urine mycotic culture, *Blastomyces* antigen, *Cryptococcus* antigen); serology for arthropod borne infections (*n* = 5), *Neospora* and *Toxoplasma* (*n* = 5) or *Distemper* (*n* = 3); commercially available serology panels for multiple infectious diseases (*n* = 2); and brain biopsy submission for fungal (*n* = 2), bacterial (*n* = 1), or *Mycoplasma* (*n* = 1) culture.

Cerebrospinal fluid was analyzed in six cases (Table 1). In three cases there was albumino-cytological dissociation, with elevated protein (32–35 mg/dL; reference range ≤ 25 mg/dL) and normal total nucleated cell count (TNCC; 1–4 cells / μL; reference range ≤ 5). In the other three cases, there was mild, mononuclear, or mixed pleocytosis (16–34 cells / μL) with elevated protein (46–80 mg/dL). No micro-organisms were identified in any case.

Besides glioma, other diseases in the differential diagnosis in the radiology report included GME/inflammatory (*n* = 3), infectious (*n* = 2), and hemorrhagic infarction (*n* = 1). Diseases included as secondary possibilities were abscess/granuloma (*n* = 3), inflammatory (*n* = 1), and hemorrhagic infarction (*n* = 1).

During the study period, 2,189 MRIs of the neurocranium were performed at Purdue and Virginia-Maryland universities. Glioma was among the top 3 MRI differential diagnoses in 14%. Of these brain MRIs supporting glioma, 148 were histologically diagnosed as glioma (surgery or necropsy), and 10 were diagnosed as granuloma. For MRIs in which glioma was a leading differential diagnosis, the ratio of histologically confirmed gliomas to granulomas was therefore 15:1.

### MRI Features

Magnetic resonance imaging was performed at Purdue, Virginia-Maryland and Illinois Universities and at two private veterinary practices. Field strengths were 1.5 T (*n* = 6), 1.0 T (*n* = 1), 0.25 T (*n* = 1), or 0.2 T (*n* = 2). In all cases, multiplanar T1W, T2W, T2W-FLAIR, and post-contrast T1W imaging was performed. In the transverse plane, T2^*^-weighted GRE (*n* = 9) and DWI (*n* = 5) were also performed.

**Figure 1:**
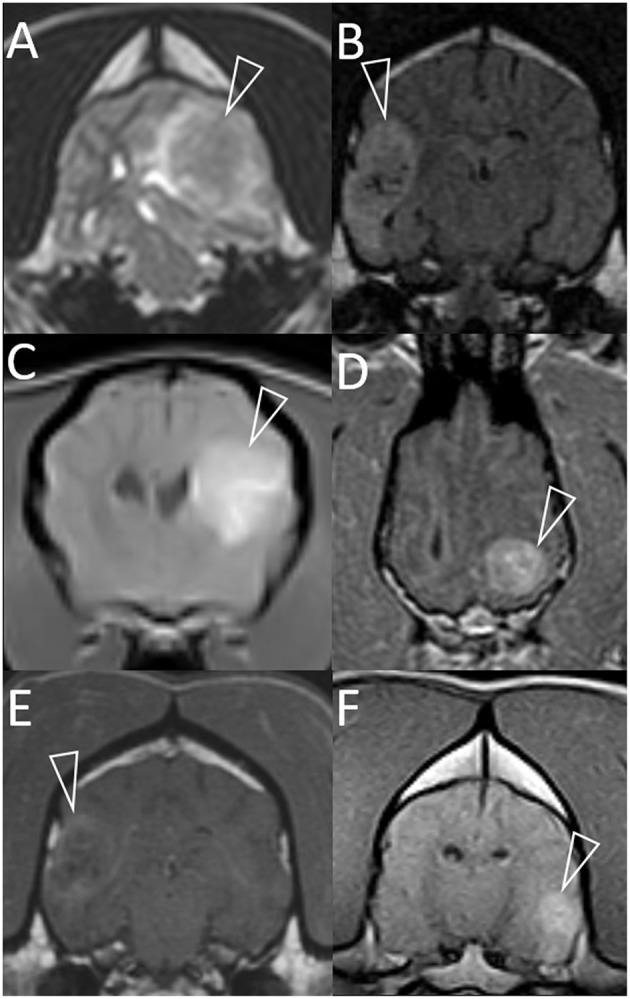
Representative magnetic resonance images of granulomas mimicking gliomas in dogs. Included are T2-weighted **(A)**, T2-weighted FLAIR **(B)**, and T1-weighted post-contrast images **(C–F)**. Images are in the transverse plane, except D (dorsal plane). In every case, a single intra-axial mass lesion (arrowheads) is present without any contralateral abnormalities. Ipsilateral lateral ventricles are distorted (all images) and variably displaced or collapsed. T2-weighted and T2-weighted FLAIR signals are heterogeneous **(A,B)**. Small, multifocal areas are FLAIR-hypointense, being isointense to CSF **(B)**. Contrast-enhancement is present **(C–F)**. The pattern varies from heterogeneous **(C,D,F)** to more peripheral **(E)**. Images represent case 1 **(C)**, case 2 **(A)**, case 3 **(D)**, case 7 **(B,E)**, and case 8 **(F)**.

**Figure 2:**
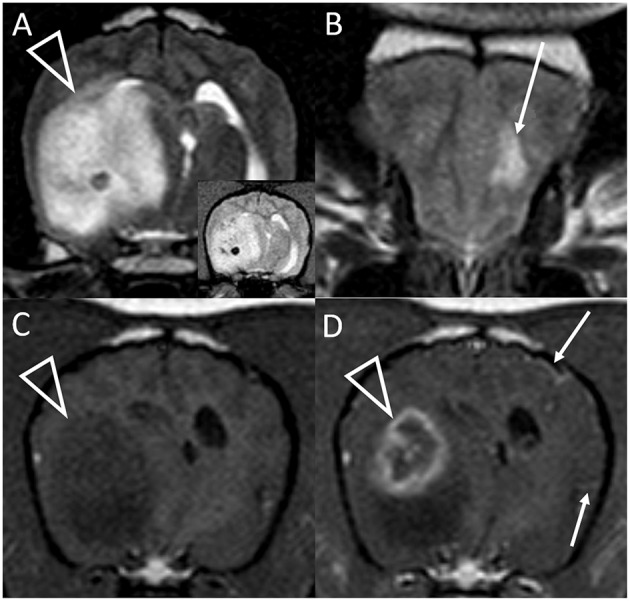
Transverse T2-weighted images at the level of the caudal thalamus **(A)** and rostral frontal lobe **(B)** and transverse pre **(C)** and post-contrast **(D)** T1-weighted images at the level of the interthalamic adhesion in a dog with granulomatous meningoencephalomyelitis (case 10). The main lesion (arrowheads) affects the right temporal lobe and thalamus causing pronounced mass effect, and is T2-hyperintense with a hypointense focus (and matching T2^*^-weighted GRE signal void, inset). In the contralateral frontal lobe is a much smaller white matter T2-hyperintensity with no mass effect (arrow, **B**). This lesion was non-contrast enhancing (not shown). There is strong, peripheral contrast-enhancement of the main lesion (arrowhead, **D**). Additionally, small foci of leptomeningeal contrast-enhancement are seen contralaterally (arrows, **D**). Although confirmed on multiplanar post-contrast imaging, these were not apparent on any other transverse image.

**Figure 3:**
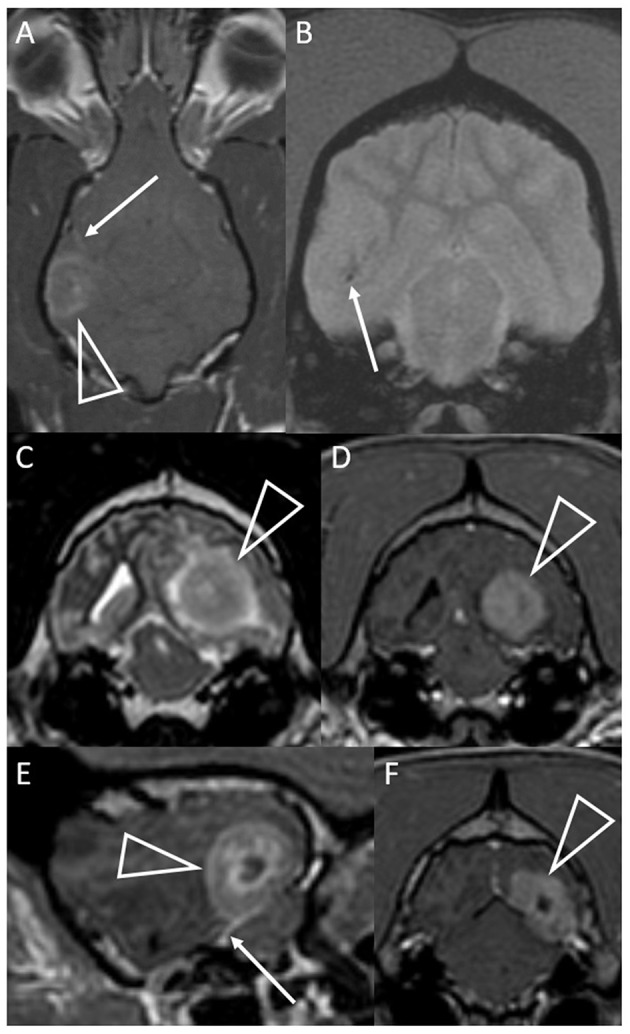
Dorsal T1-weighted post-contrast **(A)** and transverse T2^*^-weighted GRE **(B)** images of a dog with granulomatous meningoencephalomyelitis (case 7). In **(A)** an intra-axial contrast-enhancing lesion (arrowhead) makes contact with the meninges. There is leptomeningeal contrast-enhancement adjacent to the mass (arrow). In **(B)** pinpoint GRE signal voids are present in the parenchymal mass (arrow). Images of a granuloma in a dog with intra-axial and extra-axial features (case 3): transverse T2-weighted **(C)** and T1-weighted post-contrast **(D)** images at the level of the rostral occipital lobe, and T1-weighted post-contrast left parasagittal **(E)** and transverse at the level of the caudal occipital lobe **(F)**. In **(C,D)** an intra-axial mass (arrowheads) is T2-hyperintense, displays mild perilesional edema and is strongly contrast-enhancing. Mass effect is pronounced and the left cerebrum compresses the tectum. In **(E,F)** an apparently extra-axial mass (arrowheads) is continuous with the contrast-enhancement of the membranous tentorium cerebelli, where a dural tail is present (arrow). Contact with the dura is more broad-based in the transverse image, but the mass makes more parabolic contact with the dura in the parasagittal image.

The MRI characteristics are summarized in Table 2. Eight dogs had a cerebral lesion, affecting one or more of the temporal (*n* = 5), parietal (*n* = 3), occipital (*n* = 3), and frontal (*n* = 1) lobes. One lesion was temporo-thalamic and one was cerebellar. Six lesions had an unmistakably intra-axial origin (Figures 1, 2). Four lesions appeared intra-axial yet also contacted the dura mater (Figures 3A,B). One of these appeared intra-axial in some images and extra-axial in other images (Figures 3C–F).

**Table 2:** Magnetic resonance imaging characteristics of 10 canine brain granulomas.

**Magnetic resonance characteristic**	**Findings**	
Intra or extra-axial	Intra-axial (n = 6) Intra-axial with dural contact (*n* = 3)	Mixed (intra and extra-axial) (*n* = 1)
Contralateral lesions	None (*n* = 8)	Meningeal enhancement (*n* = 2) Parenchymal T2-hyperintensity (*n* = 1) Parenchymal enhancement (*n* = 1)
Mass effect	Distorted lateral ventricle(s) (*n* = 9) Midline shift (*n* = 8) Gyral flattening (*n* = 8)	Brain herniations: Sub-falcine (*n* = 6) Caudal transtentorial (*n* = 5) Caudal cerebellar (*n* = 3)
T1-weighted signal or signals	Hypointense (*n* = 6) Isointense (*n* = 5)	Homogeneous (*n* = 4) Heterogeneous (*n* = 6)
T2-weighted signal or signals	Hyperintense (*n* = 8) Isointense (*n* = 3) Hypointense (*n* = 2)	Heterogeneous (*n* = 8) Homogeneous (*n* = 2)
FLAIR (T2-weighted) signal	Heterogeneous hyperintense (*n* = 5) Hyperintense with hypointense foci (*n* = 2)	Isointense and/or hypointense (*n* = 3)
GRE (T2^*^-weighted) signal	Hyperintense, no signal voids (*n* = 7) Multifocal signal voids (*n* = 2)	Not performed (*n* = 1)
DWI signal	Hyperintense (*n* = 3) Mixed (*n* = 1) Hypointense (*n* = 1)	Not performed (*n* = 5)
Perilesional edema	Extensive (*n* = 5) Intermediate (*n* = 2)	Mild (*n* = 3)
Contrast enhancement strength	Strong (*n* = 8)	Moderate to strong (*n* = 2)
Enhancement pattern of lesion	Homogeneous (*n* = 4) Heterogeneous (*n* = 4)	Peripheral to heterogeneous (*n* = 1) Peripheral only (*n* = 1)
Enhancement of meninges	None (*n* = 1) Adjacent meninges (*n* = 6)	Extensive meningeal enhancement (*n* = 3)
Lesion margins	Well-defined, regular (*n* = 5) Well-defined, regular to irregular (*n* = 1)	Poorly defined, irregular (*n* = 4)

*All signals are presented with respect to normal gray matter*.

*DWI, diffusion weighted imaging; FLAIR, fluid attenuated inversion recovery; GRE, gradient echo*.

**Figure 4:**
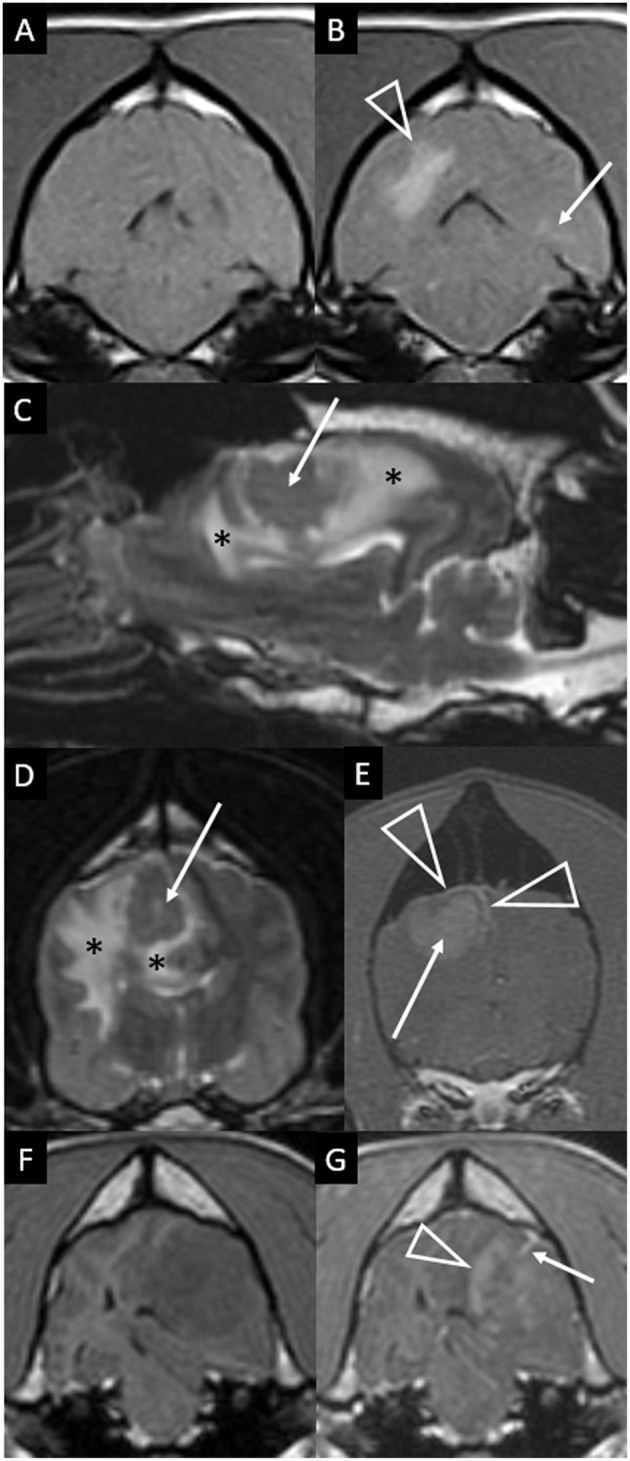
Transverse T1-weighted images pre **(A)** and post-contrast **(B)** at the level of the pons in a dog with granulomatous meningoencephalomyelitis (case 9). The caudal-most extent a large, contrast-enhancing mass within the right cerebral hemisphere is visible (arrowhead). Additionally, a small focus on parenchymal contrast-enhancement is apparent in the contralateral hemisphere (arrow). There is also high-normal to increased meningeal enhancement diffusely, especially ventral to the pons. Right parasagittal T2-weighted **(C)**, transverse T2-weighted (**D**, level of interthalamic adhesion) and transverse T1-weighted post-contrast (**E**, slightly more rostral) images of a dog (case 5) with a fungal granuloma (arrows). Perilesional edema (asterisks) affects much of the ipsilateral cerebral hemisphere. The lesion is primarily T2-isointense to normal gray matter. Markers of mass effect include midline shift, gyral flattening, and ventral displacement and collapse of the right lateral ventricle. In **(C)**, in addition to the intra-axial contrast-enhancing mass (arrow) there is concomitant enhancement of adjacent pachymeninges (arrowheads). T1-weighted pre **(F)** and post-contrast **(G)** transverse images at the level of the rostral midbrain in a dog with granulomatous meningoencephalomyelitis (case 2). A contrast-enhancing intra-axial mass lesion of the rostral occipital lobe (arrowhead) is causing severe mass effect and transtentorial herniation. There is a single focus of adjacent leptomeningeal contrast-enhancement (arrow).

Two of the ten cases had additional, much less prominent changes contralateral to the main lesion. One case had a single, small area of parenchymal T2W hyperintensity (Figure 2B). The other had a single, small area of parenchymal contrast-enhancement (Figures 4A,B).

Mass effect was prominent in all nine cerebral lesions (Table 2). The cerebellar lesion seemed to replace normal tissue more than displace it; loss of cerebellar sulci was attributed to perilesional edema.

Signal intensities are given with respect to normal gray matter, unless otherwise stated. T1W signals were often hypointense or heterogeneous (Table 2). Two cases were homogeneously T1W-isointense except a single hypointense focus, one of which was a central core nearly isointense to normal CSF. One granuloma within white matter was isointense with respect to gray matter, but conspicuously hyperintense to the surrounding normal white matter.

The T2W signal was mostly hyperintense and heterogeneous (Table 2). T2W signals generally ranged from isointense to strongly hyperintense (isointense to normal CSF). Two lesions had strongly hyperintense stipples (multiple foci isointense to normal CSF). Two fungal granulomas displayed hypointensity without a matching GRE signal void: one had a hypointense focus within an otherwise isointense lesion, the other was homogeneously mildly hypointense. Two GME lesions displayed multifocal pinpoint GRE signal voids (Figure 3B). One of these additionally had one large GRE signal void matching a focal T2W hypointensity (Figure 2A).

The FLAIR signal was often heterogeneously hyperintense (Table 2; Figure 1B). In one case, the adjacent lateral ventricle CSF was also hyperintense, supporting high CSF protein content. In two otherwise hyperintense lesions, hypointense foci matched hypointense foci on T2W imaging.

Diffusion weighted imaging was performed in five cases with various findings (Table 2). Only one case displayed strong homogeneous hyperintensity. The matching apparent diffusion coefficient map was heterogeneous without any strongly hypointense region, supporting “T2 shine through” rather than restricted diffusion.

Perilesional edema was often extensive (affecting majority of ipsilateral cerebral hemisphere/cerebellum) or intermediate (between extensive and mild). Only three cases had mild (immediately adjacent) perilesional edema.

The lesion itself displayed moderate to strong contrast-enhancement in all 10 cases (Table 2). The pattern of enhancement varied (homogeneous, heterogeneous, or peripheral) (Figures 1C–F).

The meninges displayed variable contrast-enhancement. Only one case had no meningeal enhancement. In six cases there was enhancement of the meninges adjacent to the mass. In four of these, the intra-axial mass was separated from the enhancing overlying meninges by non-enhancing parenchyma (Figure 4E) or merely made parabolic contact with the dura (Figure 3A). In the other two, enhancement of an apparently intra-axial mass was continuous with enhancement of the adjacent tentorium cerebelli (Figures 3E,F). Additional findings included a single line of leptomeningeal enhancement in two dogs (Figures 3A, 4G) or extension of the meningeal enhancement a short distance rostral to the mass in one dog.

In three cases there was more widespread meningeal enhancement. In the first, enhancement of the meninges of the ipsilateral cerebral hemisphere was considered above normal. In the second, contralateral leptomeningeal contrast-enhancement was visible on a single transverse image (Figure 2D). The third had slight, patchy, bilateral meningeal enhancement of both hemispheres, the midbrain and the ventral rhombencephalon (Figure 4B).

Margins of the lesions varied greatly from well-defined and regular to poorly defined and irregular.

## Discussion

These cases show that granuloma can “mimic” glioma on brain MRI of dogs and accordingly granuloma should not be prematurely excluded from the differential diagnosis. Along with many characteristics shared with glioma, some granulomas displayed certain MRI findings that could indicate a glioma is less likely, especially meningeal-enhancement or minor contralateral findings. Analysis of CSF is a crucial ante-mortem test, but unfortunately it does not always discriminate between glioma and granuloma. These cases also reinforce that signalment is critical in interpreting neuro-imaging.

The most significant finding of this study is that granuloma can appear indistinguishable from glioma on MRI and might be mis-diagnosed (Figure 1). This includes the classic “ring-enhancing mass” (Figure 2D) common in high-grade glioma (16). Imaging should not be over-interpreted and granuloma should not be prematurely ruled-out. Tissue biopsy remains the only definitive method to accurately diagnose these cases (20, 23). Previous studies indicate that a solitary lesion, strong contrast-enhancement, heterogeneous T2 and FLAIR signals, and mass effect are usually the key features in suspecting glioma over inflammatory and other differentials diagnoses (24–29). Other features in many of these granulomas that are identical to those reported for glioma include focal mass lesions with T1W-hypointense to isointense, T2W-isointense to hyperintense, FLAIR hyperintense signals, and so-called “cystic” lesions isointense to CSF [Figure 1B; (16, 24–31)] Similarly consistent with glioma, there were GRE signal voids supporting hemorrhage in two cases of GME (Figures 2A, 3B). GRE signal voids have not previously been reported in GME (21, 22) although hemorrhage has been suspected upon T2-weighted imaging at 7.0-Tesla imaging in one dog (32). There was also a lack of true restricted diffusion (on DWI compared with apparent diffusion coefficient maps) in all five cases in which this was performed. These findings add to the remarkable overlap of granuloma and glioma MRI characteristics (13, 33, 34). It is therefore unsurprising that in the contemporaneous radiology report from the time of diagnostic evaluation, glioma or intra-axial neoplasia was listed in the top one or two primary considerations in all cases. Infectious and inflammatory diseases were variably included as primary or secondary possibilities.

Some of these granulomas showed features that are less common in glioma but are sometimes seen. This includes more caudal location (e.g., occipital lobe being atypical for glioma), severe mass effect, extensive peri-lesional edema, and homogeneous contrast-enhancement. Although these are all possible with glioma, more common are mass effect without brain herniations, lesser peri-lesional edema and heterogeneous or peripheral enhancement (16, 24, 25, 29–31). These findings might be of use in increasing the suspicion of granuloma, while certainly retaining glioma as a possibility. Pronounced white matter tract edema and mass effect are particularly common in fungal granulomas (14, 15, 35, 36).

As shown in Figure 1, in the main these granulomas were consistent with MRI findings in glioma, with a preponderance of heterogeneous, strongly contrast-enhancing, focal intra-axial mass lesions (26). We also identified certain MRI features that may aid in the accurate recognition of granuloma, as they are not expected in glioma, and these select features are highlighted in Figures 2–4. Individual cases displayed intra-axial lesions with dural contact, mixed intra and extra-axial features, T2W-hypointensity, concomitant meningeal enhancement, or minor abnormalities of the contralateral cerebrum. Gliomas can be so superficially located that it is difficult to classify the lesion as intra or extra-axial. Nonetheless, in one study gliomas were classified as intra-axial rather than extra-axial in 153 of 155 MRI observations (25). It is atypical for glioma to have substantial dural contact or mixed intra and extra-axial features. A broad-based origination from the skull or a dural tail are usually associated with extra-axial neoplasms such as meningioma (16). In contrast, mixed intra and extra-axial features are well-described for mycotic granulomas (13, 14). Hypointensity on T2W imaging is atypical for glioma, which are up to 97% hyperintense (16, 25, 29). When present, it may relate to pathological hemorrhages. In this study, T2W-hypointensity was seen in two fungal granulomas without any GRE signal void to indicate hemorrhage as the cause. Collagenous fibers or abscess capsule formation can contribute to T2W-hypointense and isointense signals (13, 14, 37). Meningeal contrast-enhancement is not a typical feature of canine glial neoplasms (except in leptomeningeal gliomatosis, which is rare) (16, 38). Concomitant meningeal contrast-enhancement is more common in inflammatory diseases than neoplasia generally (24) and can decrease the suspicion of glioma and increase the suspicion of mycosis (14). Although diffuse meningeal enhancement is largely related to infectious and inflammatory CNS conditions (39), only one dog in the present study showed such enhancement and it was patchy (mild to none; Figure 4B). It is, however, important to note that in GME meningeal lesions are detected at the histological level but are typically not seen on MRI (21). Contrast-enhancement of the immediately overlying meninges was the most common form of meningeal-enhancement in this study, present in 6 of 10 dogs (Figure 4E). The implication of this should be further studied. Contrast-enhancement of the meninges of the whole cerebral hemisphere or even further afield as seen in three of these cases is clearly not typical for glioma and should raise the suspicion that the contrast-enhancing mass lesion is a granuloma rather than a glioma. Finally, two of these granulomas were seen as a major lesion in one cerebral hemisphere with a subtle contralateral parenchymal lesion or slight bilateral meningeal contrast-enhancement (Figures 2B, 4B). Contralateral lesions are atypical for astrocytoma or oligodendroglioma but are recognized in gliomatosis cerebri, and should also raise the suspicion of inflammatory or metastatic disease (16).

CSF analysis plays an integral role in discriminating between inflammatory and neoplastic MRI lesions (23, 27), however it was not of high utility in these cases. In brain tumors, normal CSF, albuminocytological dissociation or moderate increases in TNCC (5–50 cells/μL) are typical (40, 41). In GME, marked TNCC increases (50–900 cells/μL) are most common, but up to 10% of dogs have a normal TNCC (22). CSF findings reported for fungal granulomas include pleocytosis in 71% of cases and TNCC of 4–1150 cells/μL (13). Every CSF analysis in the current study had a TNCC in the range typical for neoplasia, below that classically seen with GME or fungal granuloma. This overlap between CSF findings in glioma, GME, and infectious granulomas is noteworthy. Unfortunately, granuloma cannot be fully excluded based on a lack of marked pleocytosis. In trying to distinguish between neoplastic and granulomatous mass lesions, there is the added difficulty that mass effect may contra-indicate CSF tap (14).

While the ability of MRI and CSF analysis to discriminate between glioma and granuloma was incomplete, signalment was very useful. None of these cases were the 5–10 years old, brachycephalic dog that is classic for glioma. The only brachycephalic dogs were a Pug and a Pekingese, two of the only brachycephalic breeds without a predisposition to glioma (42). None were Boxers, Boston Terriers or Bulldogs. Our cases included dogs both much younger and much older than the peak incidence of glioma.

These cases had scant historical, physical examination and diagnostic evidence of extracranial diseases. Over half were specifically referred by other veterinary neurologists for surgery of a suspected intracranial neoplasm. This lack of overt extraneural disease may have influenced the ranking of MRI differential diagnoses by radiologists.

A limitation of this study is that the inclusion criteria mean that these granulomas should not be considered representative of all granulomas. Rather, these cases were selected as examples of how granulomas can mimic gliomas upon MRI. The case number was small. Additionally, as this retrospective study includes cases from 2008 onwards, it is possible that the main differential diagnoses listed in the radiology report would have been different in the current era, with more readily available information regarding canine MRI interpretation and increased radiologist experience. As the majority of cases were diagnosed surgically there was a clear bias toward easily-biopsied, cerebral location. In some cases diagnosed by surgical biopsy, it is possible that a more complete diagnosis would have been rendered at necropsy. In all 10 cases there was clear histologic evidence of an inflammatory disease, however two cases responded well to antimicrobial therapy without identification of the species of micro-organism. In two other cases, the cause of inflammation could not be categorized as GME or infectious. One had histology and collagen deposition reminiscent of inflammatory pseudotumor (12, 19). The other case had received corticosteroids for 2 weeks prior to biopsy. It is possible the cause of the inflammation would have been clearer without steroid pre-treatment and the limitations of ante-mortem biopsy. A retrospective evaluation of brain biopsy in 17 dogs with encephalitis similarly revealed a specific histologic diagnosis in 82%, with encephalitis evident without a specific diagnosis in most of the other cases (43).

Future directions include further study of the MRI distinction between glioma, inflammatory lesions, and vascular lesions. These three groups of diseases are well-known to overlap upon MRI (24, 28, 34) and a sister paper to this publication focuses upon further differentiating canine glioma from vascular lesions. Functional MRI (e.g., ^1^H spectroscopy) has value in differentiating human glioma from pseudotumoral lesions (44) but was not applied in this retrospective case series.

In conclusion, granulomas can “mimic” gliomas on canine brain MRI, having many shared characteristics. The recognition of certain findings atypical for glioma (mixed intra and extra-axial features, T2W-hypointensity without apparent hemorrhage, concomitant meningeal enhancement or minor contralateral lesions) can raise the suspicion of a granuloma. CSF analysis may fail to aid in the clinical suspicion of granuloma, but signalment remains key in the interpretation of canine neuro-imaging.

## Data Availability

All datasets generated for this study are included in the manuscript and/or the supplementary files.

## Author Contributions

JR and RB: conception and design. LD and RB: acquisition of data and drafting the article. LD, AC-G, MM, DH, HH, JR, and RB: analysis and interpretation of data, revising article for intellectual content, and final approval of the completed article.

### Conflict of Interest Statement

The authors declare that the research was conducted in the absence of any commercial or financial relationships that could be construed as a potential conflict of interest.
